# On-Chip Nucleic Acid Purification Followed by ddPCR for SARS-CoV-2 Detection

**DOI:** 10.3390/bios13050517

**Published:** 2023-05-05

**Authors:** Cong Ma, Yimeng Sun, Yuhang Huang, Zehang Gao, Yaru Huang, Ikshu Pandey, Chunping Jia, Shilun Feng, Jianlong Zhao

**Affiliations:** 1 School of Information Science and Technology, ShanghaiTech University, Shanghai 201210, China; 2 State Key Laboratory of Transducer Technology, Shanghai Institute of Microsystem and Information Technology, Chinese Academy of Sciences, Shanghai 200050, China; 3 Center of Materials Science and Optoelectronics Engineering, University of Chinese Academy of Sciences, Beijing 100049, China; 4 School of Life Sciences, Shanghai Normal University, Shanghai 200235, China; 5 Department of Clinical Laboratory, The Third Affiliated Hospital of Guangzhou Medical University, Guangzhou 510150, China; 6 Department of Materials Science and Engineering, Johns Hopkins University, Baltimore, MD 21218, USA; ipandey1@jhu.edu; 7 Xiangfu Laboratory, Jiaxing 314102, China

**Keywords:** nucleic acid purification, droplet generation, ddPCR, SARS-CoV-2

## Abstract

We developed a microfluidic chip integrated with nucleic acid purification and droplet-based digital polymerase chain reaction (ddPCR) modules to realize a ‘sample-in, result-out’ infectious virus diagnosis. The whole process involved pulling magnetic beads through drops in an oil-enclosed environment. The purified nucleic acids were dispensed into microdroplets by a concentric-ring, oil–water-mixing, flow-focusing droplets generator driven under negative pressure conditions. Microdroplets were generated with good uniformity (CV = 5.8%), adjustable diameters (50–200 μm), and controllable flow rates (0–0.3 μL/s). Further verification was provided by quantitative detection of plasmids. We observed a linear correlation of R^2^ = 0.9998 in the concentration range from 10 to 10^5^ copies/μL. Finally, this chip was applied to quantify the nucleic acid concentrations of the severe acute respiratory syndrome coronavirus 2 (SARS-CoV-2). The measured nucleic acid recovery rate of 75 ± 8.8% and detection limit of 10 copies/μL proved its on-chip purification and accurate detection abilities. This chip can potentially be a valuable tool in point-of-care testing.

## 1. Introduction

The COVID-19 (Coronavirus disease 2019) outbreak has been ongoing worldwide for several years, and many diagnostic methods have been developed to detect it. The antigen test is a widely used method for pre-diagnosis thanks to its simple operation protocols and rapid result turnaround time. However, its disadvantages, including low sensitivity (10^3^ copies/μL) and false positive results, limit its application in precision medicine [1]. In contrast, quantitative real-time polymerase chain reaction can determine the relative concentration of the nucleic acids from the cycle threshold (Ct) and the standard curve obtained from samples with known concentration. It is the gold standard in detection methods because of its reliability [2].

Generally, nucleic acid purification is required before PCR to remove inhibitors prior to the amplification reaction [3]. Researchers have also tried to introduce purification-free methods to simplify the diagnosis procedures. For example, Beltran-Pavez et al. achieved extraction-free detection of SARS-CoV-2 from saliva, but their approach encountered a high percentage of false-negative problems [4]. Their work emphasized the following fact: nucleic acid purification is a prerequisite to achieve reliable and accurate detection [5], removal of inhibitors is still required [6], and it increases detection reliability by avoiding false results [7]. However, the present purification process is manual and time-consuming [8], requiring extensive washes with pipette mixing [9], and therefore needs to be implemented carefully to avoid contamination [10,11].

Some researchers have improved the detection efficiency by integrating all the purification steps on a single microfluidic chip to improve sensitivity [12]. In Ali et al.’s work, nucleic acid fragments were extracted selectively using magnetic beads and were widely used in the purification process to construct automated point-of-care systems factors [13]. This method relied on the following steps: releasing nucleic acids from samples in a lysis solution, extracting them from the contaminating solution, removing inhibitors by passing them through an oil phase and wash buffer using the magnetic beads flowing through the solutions and controlled by a magnet [14], and finally detecting the fluorescence intensities of solutions after the thermal cycling [15]. Based on this method, Juang et al. developed an oil-immersed, lossless, total-analysis system to achieve purification and detection of nucleic acids on a chip [16]. The magnetic beads entered wells via a channel controlled by a manually moved magnet under the chip. Their work proved the feasibility of microfluidic chips to improve nucleic acid purification efficiency and their potential application in point-of-care testing (POCT). In industry, some POCT devices have been applied in clinical diagnosis. Cobas Liat, the first U.S. Food and Drug Administration (FDA)-authorized POCT system, can detect SARS-CoV-2 within 20 min. However, it still suffers from false-negative results because of poor analytical sensitivities (relative to RT-PCR) [17]. The accuracy of the GeneXpert system was also lower than the Multiplex PCR assay (MPCR) in rapid diagnostics because of its qualitative detection mode [18]. These devices, to some extent, meet the requirements of POCT for simplicity, modularity, and pollution avoidance. However, the deficiencies in accuracy indicated that these qualitative detection methods used by Cobas Liat and GeneXpert were affected by PCR efficiency differences, the limitations of relative quantitative methods, and using a single result of an experiment that lacks the ability to reduce error [19].

Digital polymerase chain reaction (dPCR), proposed by Vogelstein and Kinzler [20], is an absolute quantitative nucleic acid detection method that does not need to compare with standard reference samples and does not rely on standard curves [21]. It is easy to determine the threshold for positive and negative results without requiring reference results because tens of thousands of reaction units containing no more than a single molecule can simultaneously exhibit both negative and positive results. It has the advantages of high precision, high sensitivity, and absolute quantification ability based on the Poisson distribution of nucleic acids, so it is a potential technology in early detection and monitoring [22]. Researchers have proven that the sensitivity of dPCR was determined to be equal to or greater than that of RT-qPCR [23]. In the past few years, dPCR has demonstrated its accuracy and reduced false-negative results in the ultrasensitive detection of SARS-CoV-2 and other pathogenic bacteria [24]. Droplet-based digital polymerase chain reaction (ddPCR) and cavity-based digital polymerase chain reaction (cdPCR) are the two approaches used to implement dPCR [25]. ddPCR has been widely used in past decades because of its lower cost [26], simpler fabrication process [27], and less solution waste [28] compared to cdPCR. The ddPCR is more suitable than RT-qPCR methods for determining the copy number [23]. There are some studies that have reported that ddPCR was more accurate than RT-qPCR in detecting and quantifying SARS-CoV-2 levels, especially in patients with low viral loads [29].

Yin et al. demonstrated an integrated microfluidic chip consisting of a fast nucleic acid extraction followed by a digital PCR module for POCT application [30], which realized sample-in-digital-answer-out diagnosis. Hu et al. also developed a smartphone-based digital-detection device with a rapid droplet nucleic acid isolation method for sensitive point-of-care detection [31]. Their work demonstrated the feasibility of POCT digital nucleic acid detection using magnetic-beads-based purification methods followed by dPCR.

The surface-wetting, force-driven, droplet-formation structure designed by Liu et al. [32], and the easy-to-operate co-flow step-emulsion droplet device developed by Wei et al. [28], can be used for droplet formation in the case of water–oil mixing after nucleic acid purification on a chip driven under negative pressure conditions. Their work has demonstrated the feasibility of integrating nucleic acid purification with digital PCR modules.

Based on the droplet-generation methods mentioned above, this research achieved the following goals through a newly designed microfluidic chip:

(1) The microfluidic chip was used to achieve fully enclosed nucleic acid purification, to reduce pollution, to reduce the demands on detection personnel, equipment, and the environment, and to expand the application range of nucleic acid detection;
(2) Through the innovative design of the droplet-formation structure, the on-chip nucleic acid purification and digital-detection unit were integrated on a closed chip to simplify the workflow and improve the detection efficiency and accuracy;
(3) The developed microfluidic chip was applied to detect COVID-19 nucleic acids, the chip’s performance of the was verified, and analysis and comparison were conducted to promote the application of the microfluidic chip in field detection.

Therefore, a nucleic acid purification and ddPCR integrated microfluidic chip was developed. It can achieve nucleic acid purification and quantitative analysis in an oil-enclosed environment. It uses a magnet that drives the magnetic beads to extract nucleic acid in the purification process and distributes the nucleic acids in the PCR reaction solution; it then generates separated microdroplets for ddPCR through the concentric-ring, oil–water-mixing, flow-focusing structure.

In this work, the critical component of the chip, a concentric ring, oil-water-mixing, flow-focusing droplet-generation structure, was characterized. The parameters related to droplet generation, especially the channel width at the focus point, negative pressure, and surfactant concentration, were optimized. The performance of this microfluidic chip has been demonstrated in gradient-diluted N gene plasmid sample quantification experiments. Then, the nucleic acid recovery rate was characterized by synthetic RNA fragments. Finally, accurate quantitative concentration results were generated for the SARS-CoV-2 pseudovirus.

## 2. Experimental Section

### 2.1. Chip Microfabrication

**Figure 1:**
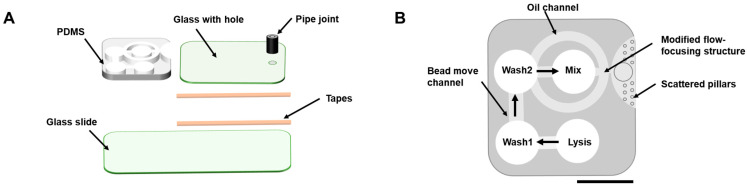
Chip structure. (**A**) Components and structure layers of the chip. (**B**) Detail of the PDMS structures.

The microfluidic chip has two main parts, as shown in Figure 1A,B. The sample-purification part has four open wells for sample lysis, washing, and mixing. The ddPCR part includes a concentric-ring, oil–water-mixing, flow-focusing structure and droplets-tilting glass cavity.

The channel and well patterns were designed using AutoCAD and then transferred using a MicroWriter maskless lithography machine (ML3, Durham Magneto Optics, Cambridge, UK) onto a 4-inch silicon wafer (MCL Electronic Materials) coated with a photoresist layer. The microfluidic block was made via standard soft lithographic methods using PDMS (DOWSIL, South Charleston, WV, USA, and SU8 3050 photoresist (Microchem, Westborough, MA, USA). The channel height was 170 ± 5 µm. The PDMS blocks were punched with four holes at locations determined by modeling structures. After bonding onto a 1 mm thick glass slide in a plasma condition, it was heated overnight in 120 °C ovens to recover hydrophobic features, ensuring that magnetic beads could move fluently and droplets could generate stably. The glass cavity was built by sticking a covere glass onto the glass slide with two pieces of 100 µm height tape. Ultraviolet curing glue was used to seal the ends of the glass cavity, the outlet tube, and the contact surface between the PDMS block and the glass cavity (Figure S1).

### 2.2. Nucleic Acid Purification

**Figure 2:**
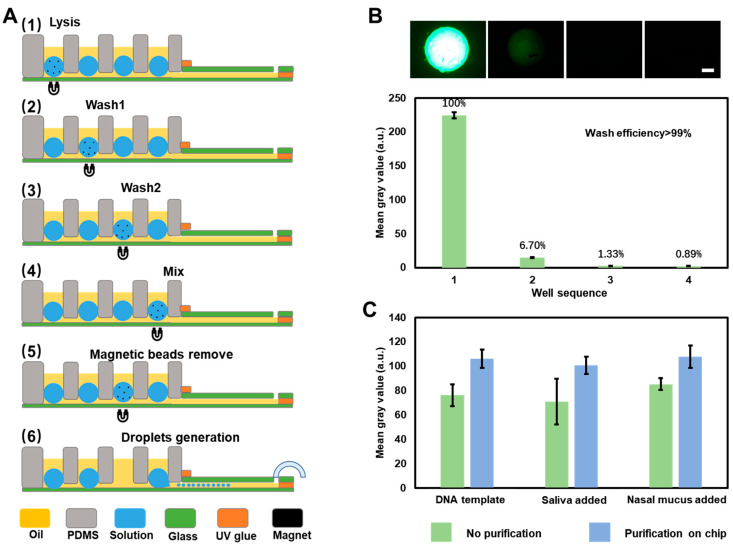
Workflow of on-chip purification processes. (**A**) The detection process, from sample loading to elution is shown as a side view in the sequence of lysis, wash 1, wash 2, and mixing in the wells from left to right. (**B**) Wash efficiency equals to the fluorescence intensity of the last drop containing the magnetic beads extracted from 20 μM FITC fluorescent dye solution in the lysis well. The scale bar represents 1 mm. (**C**) Fluorescence intensity results of the sample with interference comparison between purification on-chip and direct amplification after nucleic acid release. Error bars in the above figures represent the standard deviation based on at least three replicates of each experiment.

The chip’s purification and droplet generation processes shown in Figure 2A include the following parts: (I) The preparation steps. Fill the wells and glass cavity with oil, inject lysis reagent in well 1 (lysis well), wash solution in well 2 (wash well), rewash solutions in well 3 (wash well), and PCR reagent in well 4 (mix well) to form aqueous drops; add samples into the drop in the first well and wait 10 min for lysing (Table S1). (II) The purification steps. After the nucleic acid process is completed, pull a magnet from the first well to the fourth well under the glass slide, and introduce the magnetic beads from the lysis well to the mix well through the channel between wells. Stir the magnetic beads in each of the two wash wells and let them stay for one minute to achieve efficient washing. Finally, stir the magnetic beads for one minute and let them stay for five minutes in the mix well to elute the nucleic acid into the PCR reaction solution (Steps 1–4). (III) The amplification reaction and droplet-generation preparation steps. After the elution process, pull the magnetic beads back to wash well 2 (Step 5), where they are removed by pipette from the chip to avoid interference with the droplet generation process; (IV) Droplet generation process and amplification process (Step 6).

To further characterize the purification and detection performance, we used a sample release reagent (Sangon Biotech, Shanghai, China) to release the nucleic acid in the mix well without purificatio. We compared it with the results after the whole process on the chip (Figure S4 and Table S3). Here we detected 10^3^ copies/μL DNA template with saliva and nasal mucus to simulate actual nasopharyngeal swab samples. The results without and with purification are shown in Figure 2C and indicate that lysing the sample directly in the PCR reaction reagent brought inhibitors in an amplification reaction, which caused a lower end-point fluorescence intensity for the drop. This experiment proved that the purification step improved fluorescence intensity by 26%, improving the fluorescence difference between positive and negative results. In addition, the purified results reduced the margin of error by 52% compared to the unpurified results. This inhibitor phenomenon was also reported by N. Vasudevan et al. [33]. The fact that absolute quantification measurement results in droplets can reduce this distraction was also pointed out in their work.

### 2.3. Droplet Generation

Prior to droplet generation in the chip, some preparation is required. Hydrophobicity is crucial for droplet generation and avoiding droplet rupture. The contact angles of slides increased clearly in the hydrophobicity of the bottom glass slide and cover glass after treatments. Sigmacoat (Sigma-Aldrich, St. Louis, MO, USA) was selected as the hydrophobic treatment reagent because its coat is formed by chemical reaction and shows good durability throughout the experiment. Surfactants also play an important role in avoiding droplet fusion (Figure S2). Mineral oil (Sigma-Aldrich, USA) was homogenized by an ultrasonic bath after adding EM90 (Degussa, Frankfurt, Germany) 3.59%, Triton X100 (Sigma-Aldrich, USA) 0.13%, and Span 80 (Sigma-Aldrich, USA) 0.79% in mass fraction to make the droplet-generation oil.

In the droplet-generation experiment, a negative pressure, set using an air pressure valve range from −25 kPa to −40 kPa, was found suitable to generate pure water droplets. Pressure lower than −5 kPa cannot pull the solution drop from the last well to the focusing point in the channel. On the other hand, negative pressure exceeding a certain threshold will lead to a continuous flow because the higher-flow-rate aqueous phase cannot be discretized by oil flow.

**Figure 3:**
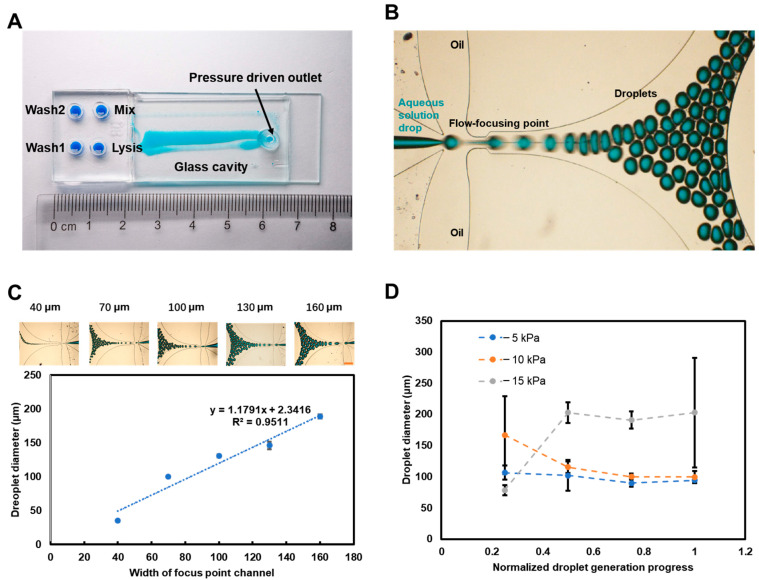
Dropletgeneration performance test. (**A**) Photograph of the microfluidic chip filled with a millimeter-scale drop in four wells and generated micrometer-scale droplets in a glass cavity. (**B**) Blue-dye-solution droplet-generation process in the concentric-ring, oil-water-mixing, flow-focusing structure. (**C**) The linear correlation between the droplet diameter and the width in 165 μm channel-height chip driven by −25 kPa pressure. The insert images show that the droplet diameter increases with increases in width. Scale bar = 500 μm. (**D**) The variation in diameter of 1% Tween 20 solution droplets during the whole droplet-generation process in the chip with 80 μm width driven under different negative pressures. Error bars in the above figures represent the standard deviation based on at least 3 replicates of each experiment.

In the following experiments, the width of the aqueous phase channel at the focusing point, the surfactant concentration, and the negative pressure value were the main factors determining whether the droplets could be formed successfully. At first, chips with different widths of channels at the flow-focusing point were used to generate droplets. The results, shown in Figure 3C, revealed the linear relationship between the channel width and the diameter of the droplet. These results proved that this concentric-ring, oil–water-mixing, flow-focusing structure could generate different size droplets by simply setting the channel width of the aqueous phase at the focus point.

**Figure 4:**
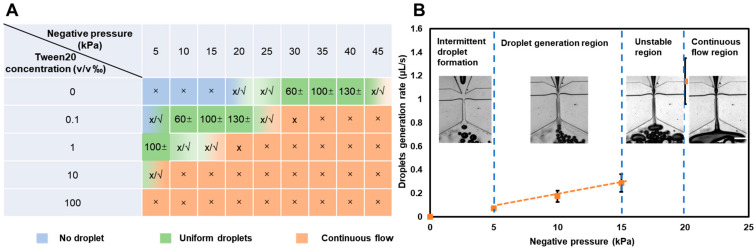
Droplet formation test. (**A**) Negative pressure and surfactant concentration effects on droplet generation. The numbers in the table are the mean value of the droplet diameter. ’x’ represents droplet formation failed, and ’√’ represents droplet formation succeed. (**B**) Droplet-generation change with the negative-pressure change for 1% Tween 20 solutions driven under −5 kPa pressure. The insert images show the droplet-generation process under the corresponding pressure range. Scale bar = 500 μm.

The surfactant is an essential material in the reagent for avoiding droplet fusion [34]. The related factors, including the driven negative pressure and surfactant concentration, were characterized to find the droplet-generation condition (Figure S3). The results revealed that the existence of surfactant reduced the required driven negative pressure by shifting the pressure from a high range to a range lower than −25 kPa. However, excessive surfactant concentration in the solution led to a failure to generate droplets (Figure 4A), which was because the surfactant decreased the solution’s surface tension and increased the aqueous phase flow, resulting in insufficient shear force being provided by a fixed oil flow, which then cannot separate the aqueous flow into discrete droplets.

The droplet-generation experiment was performed using the optimized chip in Figure 3A. The statistical analysis results, as shown in Figure 3B,C, indicated that the linear correlation between the diameter of pure water droplets and slit width was y = 1.1791x + 2.3416 in the chip with 165 μm channel height under the condition of being driven by −25 kPa pressure. This result showed that droplets of different sizes can be obtained simply by changing the width of the water channel.

The results of droplets’ diameter change over time during the generation process are shown in Figure 3D, and these indicated the following features for the solution with 1% Tween 20 (Sigma-Aldrich, USA) surfactant: (1) The droplet generation at the midpoint of the entire process is more stable than at the start and end points. Especially at the endpoint, the drop will pass through the flow-focusing point fluently without splitting because the size of the remaining drop is too small to be restricted in the mix well. (2) The droplet-generation process driven under pressures ranging from −5 to −15 kPa is more stable than when driven with lower and higher pressures. Drivenunder pressure ranging from 0 to −5 kPa, the aqueous-solution drop only intermittently reached the flow-focusing point due to surface tension pulling the deformed drop tip back. This occurred when the aqueous solution was drawn to the flow-focusing point, and the split of the aqueous phase with increasing flow rate became difficult due to a limited shearing force of oil. (3) The diameter of the droplet was about 100 μm, controlled by the 80 μm width channel and driven under −5 and −10 kPa pressure at a stable state, but increased to about 200 μm at the beginning. The drop movement could lead to this phenomenon, which was generated from the flow-focusing point to the converging channel in the flow direction.

The effect of surfactants on droplet generation indicated that the chip may have compatibility problems when PCR reagents contain surfactants with different concentrations. The following three approaches were used in our experiment to solve this problem: (1) Reduce the surfactant concentration in the oil or use pure mineral oil. (2) Set a stable PCR mix reagent and add different upstream primers, downstream primers, and probe reagents. The small volume fraction of the primers and probes for other target genes have little effect on the overall PCR reagent, so they ensure this chip can be used in different detection applications. (3) Reduce the mix well diameter from 5 mm to 2 mm to maintain the drop formation of a high-surfactant-concentration solution, which avoids having the solution flow out of control at the bottom of the well.

The fluctuation of droplet diameters during the droplet generation process reduces the accuracy of the quantification results. However, the number of these nonuniform droplets will not exceed 1%, so this fluctuation has only a limited impact on the quantitative results. This will also ensure the qualitative results, even though the detection concentration will be lower than the real concentration if the uniform droplets contain more than one nucleic acid. However, the quantitative results of dPCR for low-concentration nucleic acid have a large margin of error originally due to the very small number of positive droplets.

In summary, this chip offered good droplet-generation performance under suitable pressures. It can dispense a 5 μL solution drop into 10,000 monodispersed 100 μm diameter droplets, with a flow rate ranging from 0 to 0.3 μL/s when driven under negative pressure. Its corresponding advantages include eliminating the two channels in the traditional positive-drive-pressure flow-focusing structure in favor of merely one, and avoiding channel absorption for the sample solution. It also exhibits robustness when encountering the risk of being blocked by impurities like dust because the length of the micrometer-scale narrow channel in the whole chip is smaller than 2 mm. In such a case, the droplets can still be generated stably even if one of the oil channels is blocked. Moreover, this method avoids bubbles due to the oil phase and the aqueous phase arriving at the flow-focusing point at different times by prefilling the oil.

### 2.4. Detection Verification

The chip’s ability to quantify the plasmids with the SARS-CoV-2 gene was verified by adding only PCR reaction solution to the fourth well (Table S2). The PUC57 plasmid with N gene sequence was procured from Sangon Biotechnology (Shanghai, China). After the droplet was generated, the chip was put on PCR thermocycling amplifiers (Eppendorf, Hamburg, Germany), with which wet gloves and metal containers were required to avoid droplet fusion (Table S6). Then, an IX51 microscope with a DP80 camera (Olympus, Tokyo, Japan) was used to take pictures of the droplet’s storage cavity after the amplification reaction. ImageJ software was used to analyze all fluorescence images to separate positive droplets from negative droplets.

**Figure 5:**
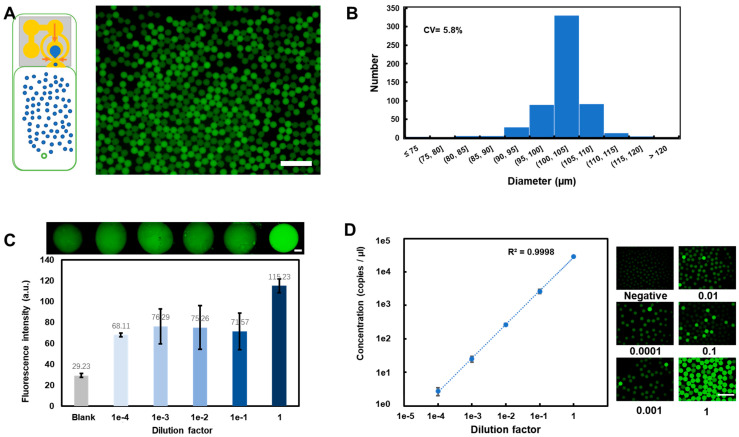
Chip verification. (**A**) Left: work mode without purification process; right: fluorescence images of the droplets for 100,000 copies/μL after PCR. (**B**) The diameter distribution of droplets. (**C**) Qualitative gradient dilution results of 100,000 copies/μL N gene DNA template by measuring the fluorescence intensity of drop in the last well. (**D**) The linear correlation (blue dashed line) between the measured concentrations and five tenfold gradients dilution factor of about 100,000 copies/μL N gene DNA molecules. The data are expressed as mean and standard deviation based on at least three replicates of each experiment. All scale bars represent 500 μm.

In the following experiments, about 10^5^ copies/μL N gene DNA templates were diluted in five gradients to verify the detection accuracy of the chip. Figure 5A,B shows the fluorescence picture analysis. It shows that the droplet’s variable coefficient size distribution was 5.8%. These results indicate good stability during droplet generation and heating, which determines the precision of the quantification results. Qualification was also implemented simultaneously in this chip by leaving some reagent in the mix well during droplet generation. The larger drop sizes in the mix well can be identified using optical detection devices, making it suitable for different applications and instruments. The performance of the qualitative identification of millimeter-scale drops is shown in Figure 5C using fluorescence images and the mean gray value of drops after 45 thermal cycles. The mean gray values of drops were used to distinguish negative and positive samples. These results confirmed that the qualitative result had a clear difference between positive and negative samples but also emphasized that it is difficult to quantify the concentration of nucleic molecules due to the deviation caused by many factors such as drop deformation, amplification efficiency, thermocycler number, etc. Results in Figure 5D show excellent agreement with the dilution factors in the 10^−5^~10 range for about 10^5^ copies/μL nucleic acid in all reactions with a linear fit curve (R^2^ = 0.9998). These good quantification results emphasized the role of ddPCR in precise diagnosis (Table S4).

### 2.5. SARS-CoV-2 Diagnosis

**Figure 6:**
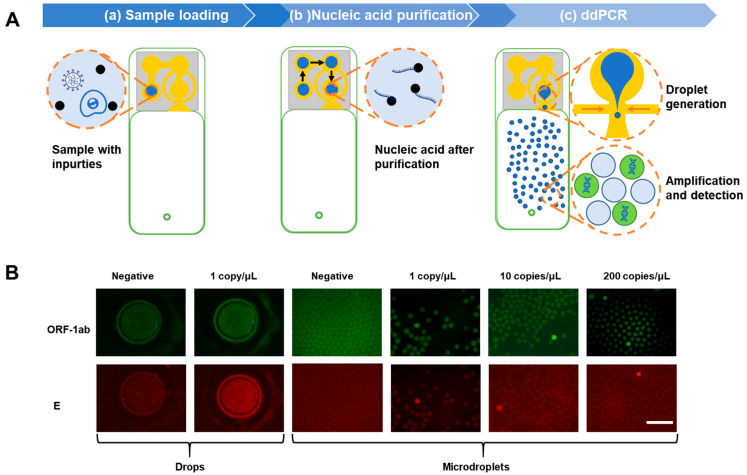
SARS-CoV-2 on-chip diagnosis. (**A**) On-chip workflow from nucleic acid purification to digital detection is shown as the top view. (**B**) The detection-limit test of 1 copy/μL of SARS-CoV-2 pseudovirus shows qualitative results from the millimeter-scale drop and quantitative results from microdroplets. All scale bars represent 500 μm.

The pseudovirus was processed and detected in this chip to simulate the SARS-CoV-2 diagnosis. After the purification process of SARS-CoV-2 on a chip, as mentioned above, the outlet of the chip was connected to a negative pressure system, and the pressure regulator was set at a suitable value. The PCR mixture solution drop was cut into microdroplets by flowing oil when it passed through the flow-focusing structure when driven under negative pressure (Figure 6A). The droplets were stored in the cavity for 45 thermal cycles (Table S7) and the following detection.

The images shown in Figure 6 only represent the part of the positive droplets found in the microscope field because low-concentration positive results cannot show in gradient in images with hundreds of droplets. The final quantitative results in this work were calculated by statistical results from more images than are shown in these figures (Table S5). The micron-sized, microdroplet-based quantitative diagnosis method compared with the millimeter-sized-drops qualitative diagnosis method is shown in Figure 6B. It confirms the reliability and accuracy of this ddPCR chip in detecting 10 copies/μL (100% repeatability). The chip also exhibited the potential of ddPCR for 1 copy/μL sample diagnosis with an 80% probability, and 60% probability of single-drop qualitative detection in the mix well. These results indicated the following advantages of droplet digital quantitative detection: higher repeatability in low-concentration detection, absolute count and calculation without comparing to negative or standard reference, and ease of distinguishing the positive results from surrounding negative droplets.

With the help of the quantification ability of the ddPCR, the nucleic acid recovery rate of the purification process was measured by comparing the measured nucleic acid concentration result for RNA fragments (RNA with and without purification). Then, 200 copies/μL of SARS-CoV-2 pseudovirus (Bangdesheng Biotechnology, Guangzhou, China), the highest-concentration pseudovirus in the laboratory, were quantified with nucleic acid release and detection kits (Shengxiang Biotechnology, Shanghai, China). The results in Table 1 indicate that magnetic beads could extract 75 ± 8.8% nucleic acid from the solution in the first well. This efficiency improved upon previous work by other researchers, such as Hu et al. [31] (recovery = 75%) and Carvalho et al. [35] (recovery = 42%). Other nucleic molecules may be lost during the capture, wash, and elution process. This recovery rate can calculate a more accurate concentration of nucleic molecules in the primary sample. However, a pseudovirus nucleic acid recovery rate of 66 ± 3.8% was also measured in our exper. This which could have been due to non-ideal lysis efficiency, which is limited by lysis time and reagent.

## 3. Conclusions

A nucleic acid purification and ddPCR integrated microfluidic chip was developed in this work. Its primary component, a concentric-ring, oil-water-mixing, flow-focusing structure for microdroplets generation, was designed and optimized. This structure can generate many picoliter-sized, uniform, monodispersed droplets from micrometer-scale drops in oil when driven under negative pressure. This structure highlights the feasibility of the whole ddPCR process being integrated with a sample-purification module on a single chip. This structure also provides a novel droplet-generation method for digital detection after oil-enclosed purification, which avoids needing to extract the solution drop to implement PCR in another chip. It transforms the endpoint single-drop qualitative analysis into absolute digital quantification. The chip’s advantages include being simple and easy to operate, no reagent waste, high integration level, prevention of aerosol contamination, and high sensitivity. It achieved higher-accuracy detection by replacing the qualitative detection of the currently used on-chip purification and detection chip, and it improved the integration level of nucleic acid detection by integrating purification ddPCR detection. On this basis, a 66 ± 3.8% recovery rate of nucleic acid and an available detection limit of 10 copies/μL in SARS-CoV-2 pseudovirus detection were characterized. When used for 1 copy/μL sample detection, this chip has an 80% detection probability for positive samples.

Compared with other works using continuous flow PCR microfluidic chips [36] and digital microfluidic platforms with magnetic beads [37], our chip takes longer to work, but it is easier and less costly to process because it does not include electrodes. It is also more accurate because of the quantitative results. Yin et al. reported a rapid nucleic acid purification and digital-detection method for on-site, real-time detection. Still, it used a microcavity as the reaction unitwhich is difficult to separate the water phase in oil to fill the microcavity. Our chip showed the advantages of convenience and efficiency, simple processing, and high integration [38]. The results obtained in this study show higher sensitivity than that of RT-PCR methods and a lower than the 0.1–1 copies/μL average detection limit of dPCR methods of previous studies [39]. The unsatisfactory detection limit may be due to the unsatisfactory purification effect on the chip compared to professional nucleic acid purification equipment. The results in this experiment have higher accuracy and better linear correlation compared with the multi-biomarker colorimetric detection on-chip RT-LAMP method [40]. However, miniaturized equipment for droplet detection needs to be further studied to meet POCT detection requirements, among which the most important is to reduce the volume of optical detection equipment. Optical fibers [41] and a lensless microscopic imaging technique [42] could be a potential miniaturization solution.

After the abovementioned experimental verification and comparison of results, we believe that this chip could be a powerful supplement to the present ddPCR and oil-based nucleic acid purification microfluidic chips because of the following advantages: (1) the potential to apply automation pressure with a magnetically driven method; (2) minimal cross-contamination in oil-sealed conditions; (3) the high accuracy and sensitivity of digital quantification; and (4) the ease of expelling the droplets from the chip to determine the amplification product. It could be a valuable digital microfluidic chip for contamination-free, accurate, automated detection in POCT applications.

## 4. Discussion and Outlook

The remaining challenges and opportunities for improvement for this chip are as follows: (1) integrating multi-chips into the system for high-throughput detection, which could be achieved with multi-channels design in the microfluidic chip; coupling the magnetically controlled motion system to reduce manpower requirements; replacing the manually controlled pressure regulator with automated, programmed pressure systems to improve the automation level; (2) optimizing the droplet-generation structure to be more stable in the whole droplet-generation process; applying rapid droplet digital PCR reactions to reduce the time required; (3) implementing an injection-molding fabrication technique to reduce the cost and complexity of present chip production; (4) improving upon the glass cavity because it contains sparsely distributed droplets and takes up substantial space; designing a dual-layer chip in which the glass cavity under the purification module will comprise more than half the chip area; and (5) further optimizing the structure to avoid small-size satellite droplets being generated accidentally along with normal droplets, as shown in Figure S3.

## Figures and Tables

**Table 1:** RNA quantification results.

Sample Type	Primary Concentration (Copies/μL)	Concentration (Copies/μL)	Recovery (%)
Synthetic RNA fragments	279 ± 16	226 ± 14	75 ± 8.8
Pseudovirus	200	133 ± 7	66 ± 3.8
	10	4 ± 1	40 ± 25.0

## Data Availability

Not applicable.

## Supplementary Materials

The following supporting information can be downloaded at: https://www.mdpi.com/article/10.3390/bios13050517/s1, Figure S1: CAD design drawing of lithography mask and fabrication process of the chip; Figure S2: Comparison of hydrophobic performance of the glass slides after different hydrophobic treatments, and two kinds of reagent solution hydrophobic performance on the glass slide; Figure S3: Droplet formation of aqueous reagents with different surfactants; Table S1: The reagents composition; Table S2: Primer and MGB probe sequence for the ORF1ab, N, and E genes; Figure S4: Fluorescence intensity drops compared to amplification after lysis and amplification after purification on the chip; Table S3: Fluorescence intensity of drops comparison after different process; Table S4: Quantitative concentration of plasmid with N gene; Table S5: Quantitative concentration of synthetic RNA and pseudovirus; Table S6: PCR amplification procedure of DNA plasmid; Table S7: PCR amplification procedure of Synthetic RNA fragment.
