# Supplementary material for: On-Chip Nucleic Acid Purification Followed by ddPCR for SARS-CoV-2 Detection
Source: Biosensors (Basel). 2023 May 5;13(5):517. doi: 10.3390/bios13050517 (PMC10216157; doi:10.3390/bios13050517)
Supplement: Supplementary file 1 [file biosensors-13-00517-s001.zip › biosensors-2342092-supplementary.pdf]

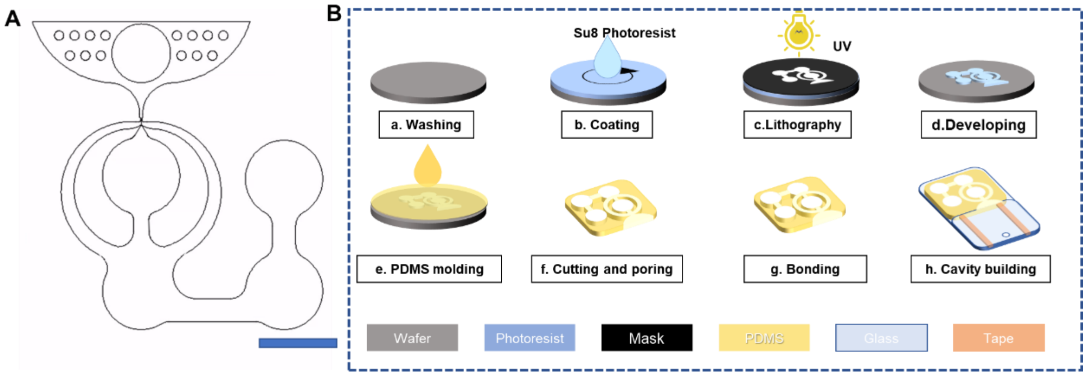

Figure S1. (A) CAD design drawing of lithography mask. Scale bar=5 mm (B) Fabrication process of the chip

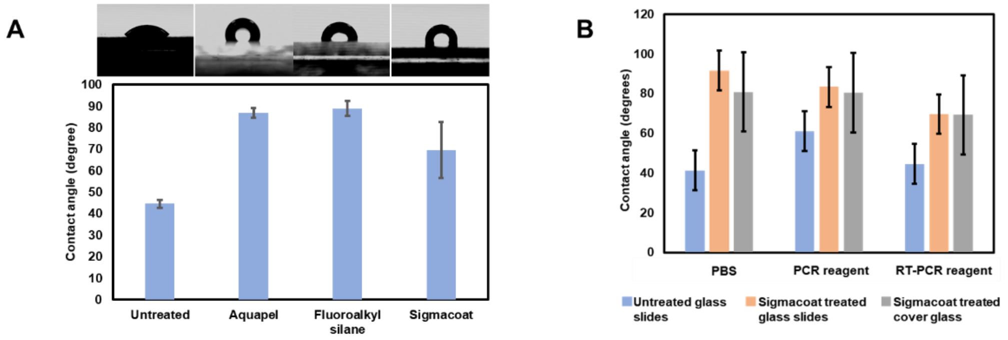

Figure S2. (A) Comparison of hydrophobic performance of the glass slides after different hydrophobic treatments. (B)Two kinds of reagent solution hydrophobic performance on the glass slide reveal the reason for various droplet generation performances, which indicated the inverse transcription PCR mixture for RNA detection has a higher surfactant concentration than PCR for plasmid detection.

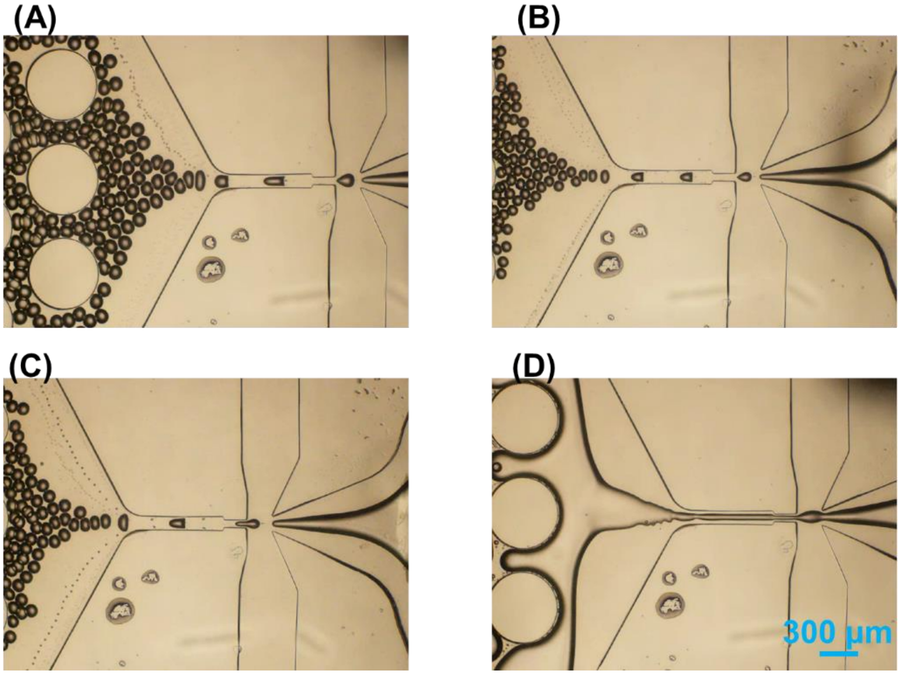

Figure S3 droplet formation of aqueous reagents with different surfactants: (A) pure water without surface activity; (B) 0.01% tween20 reagent; (C) 0.1% tween20 reagent; (D) 1% tween20 reagent

Table S1. The reagents composition

Solution type	Component	Proportion (v/v %)
Lysis solution	Lysis solution	65
	Protein K	5
	Magnetic beads	10
	2019-nCOV pseudovirus/ RNA	20
Wash solution 1	60% Ethyl alcohol	100
Wash solution 2	PEG-8000	13
	1mM $MgCl_2$	87
PCR solution for pseudovirus	Mix	52
	Reverse transcriptase	8
	DEPC water	40
PCR solution for N gene plasmid	DEPC water	28
	Mix	50
	10μM forward primer	4.5
	10μM reverse primer	4.5
	10μM TaqMan probe	3
	pUC57 plasmid	10

Table S2. Primer and MGB probe sequence for the ORF1ab, N, and E genes

Gene	Reagent	Sequence（5’-3’）
ORF1ab<br>CCATGCCTAACATGCTTAGAATATGGCCTCACTTGTTCTTGCTCGCAAACATACAACGTGTTGTAGCTTGTCACACCGTTTCTATAGATTAGCTAATGAGTGTGCTCAAGTATTGAGTGAAATGGTCATGTGTGGCGGTTCACTATATGTTAAACCAGGTGGAACCTCATCAGGAGATGCCACAACTGCTTATGCTAATAGTGTTTTTAACATTTGTCAAGCTGTCACGGCCAATGTTAATGCACTTTTATCTACTGATGGTAACAAAATTGCCGATAAGTATGTCCGCAATTT	Forward primer	TAGCTAATGAGTGTGCTCAAGTATT
	Reverse primer	GTTGTGGCATCTCCTGATGAG
	TaqMan probe	FAM-TGGTCATGTGTGGCGGTTCACTAT-MGB
E-Gene<br>CTTTGTAAGCACAAGCTGATGAGTACGAACTTATGTACTCATTCGTTTCGGAAGAGACAGGTACGTTAATAGTTAATAGCGTACTTCTTTTTCTTGCTTTCGTGGTATTCTTGCTAGTTACACTAGCCATCCTTACTGCGCTTCGATTGTGTGCGTACTGCTGCAATATTGTTAACGTGAGTCTTGTAAAACCTTCTTTTTACGTTTA	Forward primer	ACAGGTACGTTAATAGTTAATAGCGT
	Reverse primer	ACAGGTACGTTAATAGTTAATAGCGT
	TaqMan probe	ROX-ACACTAGCCATCCTTACTGCGCTTCG-MGB
N-Gene<br>GCTGGACTTCCCTATGGTGCTAACAAAGACGGCATCATATGGGTTGCAACTGAGGGAGCCTTGAATACACCAAAAGATCACATTGGCACCCGCAATCCTGCTAACAATGCTGCAATCGTGCTACAACTTCCTCAAGGAACAACATTGCCAAAAGGCTTCTACGCAGAAGGGAGCAGAGGCGGCAGTCAAGCCTCTTCTCGTTCCTCATCACGTAGTCGCAACAGTTC	Forward primer	TGAGGGAGCCTTGAATACACC
	Reverse primer	GGAAGTTGTAGCACGATTGCA
	TaqMan probe	HEX-ATTGGCACCCGCAATCCTGCTAAC-MGB

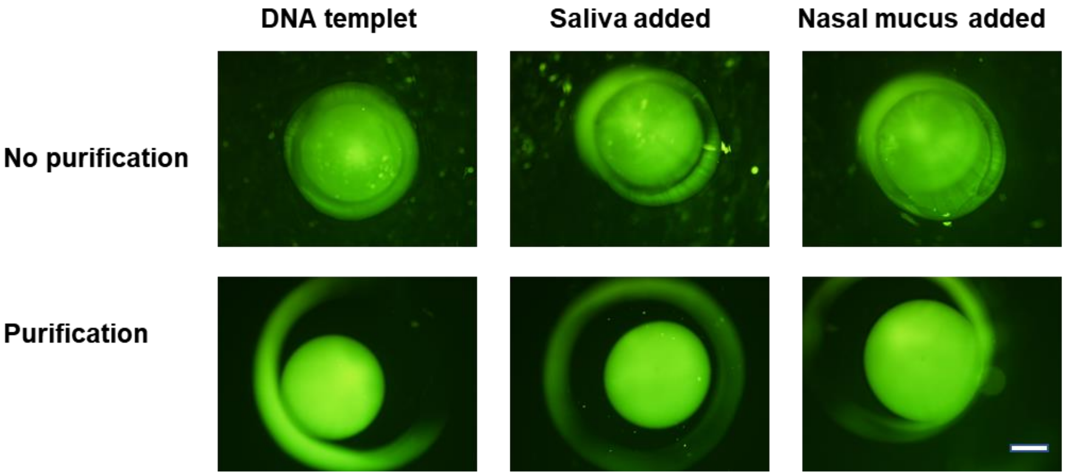

Figure S4. Fluorescence intensity drops compared to amplification after lysis and amplification after purification on the chip. The detection object is $10^{\wedge 3}$ copies/μL DNA plasmid template with the N gene of SARS-CoV-2. Scale bar=1mm.

Table S3. Fluorescence intensity of drops comparison after different process

Sample type	No purification	Mean	Standard deviation	Purification	Mean	Standard deviation
DNA templet	100.52<br>68.38<br>88.88	85.93	13.29	96.66<br>114.93<br>106.27	105.95	7.46
Saliva added	71.43<br>85.04<br>83.47	79.98	6.08	91.70<br>109.02<br>100.74	100.48	100.48
Nasal mucus added	44.20<br>90.07<br>80.48	71.58	19.75	100.72<br>102.04<br>120.76	107.84	9.15

Table S4. Quantitative concentration of plasmid with N gene

Dilution factor	Measured concentration (copies/μL)	Average value (copies/μL)
	3.50	
0.0001	2.00	2.83
	3.00	
	31.67	
0.001	22.33	24.94
	20.82	
	243.55	
0.01	284.37	267.33
	274.07	
	3001.25	
0.1	2261.56	2753.86
	2998.77	
	28168.43	
1	27535.70	28661.07
	30279.08	

Table S5. Quantitative concentration of synthetic RNA and pseudovirus

Sample type	Process	Norminal concentration	Measured concentration	Mean	Standard deviation	Recovery (%)	Relative standard deviation (%)
Synthetic RNA	No purification	Blank	283				
			259	280	16		
			297			75	0.08
	Simulated Purification		226				
			192	209	14		
			210				
Pseudovirus	Purification		126				
		200	139	132	5	66	3.78
			131				
			5				
		10	3	4	1	40	25
			3				

TableS6 PCR amplification procedure of DNA plasmid

Temperature (℃)	Time (s)	Cycle number
95	600	1
95	10	45
58	45	

TableS7 PCR amplification procedure of Synthetic RNA fragment

Temperature (℃)	Time (s)	Cycle number
50	1800	1
95	60	1
95	15	45
58	30	
